# Flash Gas Chromatography in Tandem with Chemometrics: A Rapid Screening Tool for Quality Grades of Virgin Olive Oils

**DOI:** 10.3390/foods9070862

**Published:** 2020-07-02

**Authors:** Sara Barbieri, Chiara Cevoli, Alessandra Bendini, Beatriz Quintanilla-Casas, Diego Luis García-González, Tullia Gallina Toschi

**Affiliations:** 1 Department of Agricultural and Food Science, Alma Mater Studiorum-Università di Bologna, 47521 Cesena, Italy; sara.barbieri@unibo.it (S.B.); chiara.cevoli3@unibo.it (C.C.); tullia.gallinatoschi@unibo.it (T.G.T.); 2 Department de Nutrició, Ciències de l’Alimentació i Gastronomia, Campus De l’Alimentació Torribera, Facultat de Farmàcia i Ciències de l’Alimentació, Universitat de Barcelona, 08921 Santa Coloma de Gramenet, Spain; beatrizquintanilla@ub.edu; 3 Institut de Recerca en Nutrició i Seguretat Alimentària (INSA-UB), Universitat de Barcelona (UB), 08921 Santa Coloma de Gramenet, Spain; 4 Instituto de la Grasa (CSIC), 41013 Sevilla, Spain; dlgarcia@ig.csic.es

**Keywords:** virgin olive oil, quality, volatile compounds, sensory analysis, chemometrics

## Abstract

This research aims to develop a classification model based on untargeted elaboration of volatile fraction fingerprints of virgin olive oils (*n* = 331) analyzed by flash gas chromatography to predict the commercial category of samples (extra virgin olive oil, EVOO; virgin olive oil, VOO; lampante olive oil, LOO). The raw data related to volatile profiles were considered as independent variables, while the quality grades provided by sensory assessment were defined as a reference parameter. This data matrix was elaborated using the linear technique partial least squares-discriminant analysis (PLS-DA), applying, in sequence, two sequential classification models with two categories (EVOO vs. no-EVOO followed by VOO vs. LOO and LOO vs. no-LOO followed by VOO vs. EVOO). The results from this large set of samples provide satisfactory percentages of correctly classified samples, ranging from 72% to 85%, in external validation. This confirms the reliability of this approach in rapid screening of quality grades and that it represents a valid solution for supporting sensory panels, increasing the efficiency of the controls, and also applicable to the industrial sector.

## 1. Introduction

The official methodology for sensory evaluation of virgin olive oils (VOOs), known as a panel test, is a fundamental tool to assess the quality of products that cannot be replaced by instrumental methods, considering that the overall and complex perceptual attributes (e.g., fruity and defects) are the indicators of the quality of VOOs. Despite its proven effectiveness in evaluating the quality grades of samples, tested in EU countries since 1991 [1,2], the scientific community has highlighted some drawbacks on its application that are mainly related to the following: (i) the reproducibility of results among different panels; (ii) critical attribution of the category when, e.g., a defect is borderline; (iii) costs, assessor fatigue and other limitations associated with a method working with humans.

Specifically, according to decisions taken at International Olive Council (IOC) level, the Reg. (EU) 1348/2013 [3] recommends the number of oils to be assessed by the sensory panels, fixing a maximum number of four samples at each session. Moreover, a maximum of three sessions per day is specified, to leave enough time between a session and another, thus avoiding the contrast effect that could be produced by immediately tasting sequences of samples. These specifications strongly limit the number of samples that can be assessed by one panel per day. On the other hand, to enhance panel skills in recognizing, identifying, and quantifying sensory attributes, the introduction of new artificial reference materials (obtained by chemical or biotechnological approaches), could improve the proficiency of the individual panels and their global alignment by overcoming some limitations associated with a natural matrix (e.g., limited amounts available, difficultly obtaining, low homogeneity year by year) and offering advantages such as preparation in each laboratory, reproducibility over time, possibility of purchase, and therefore their availability for the market.

In this context, the development of an instrumental method for rapid screening of quality grades of samples (extra virgin olive oil, EVOO; virgin olive oil, VOO; lampante olive oil, LOO) could represent a solution to support sensory panels (particularly for large private industries), decreasing their daily work by reducing the samples that need to be assessed (e.g., by excluding those definitely compliant), with a consequent increase in the efficiency of quality controls and reducing the number of samples that need to be controlled.

In this way, improvement of the activity of sensory panels, whose work remains central to ensuring the quality of the product, would be achieved by focusing sensory analysis only on uncertain samples (i.e., borderline oils between two product categories that can be the object of disagreement among panels).

It is well known that volatile compounds are crucial to determine VOO quality and that they are responsible for the different VOO sensory profiles [4,5,6]; their determination in a rapid way (e.g., screening method) could support sensory analysis and represents one of the current challenges in the olive oil sector where fast, accurate, and easy-to-use approaches providing real-time results are required.

Recently, different analytical techniques combined with chemometric statistical approaches have been proposed to predict sensory information [7,8,9].

Alongside the traditional techniques (targeted) in which specific and selected molecular markers are monitored during the analysis to assess the presence or absence of compounds and their quantification, untargeted analyses, based on a holistic approach and able to provide information such as a spectral fingerprint, giving a simplified and overall picture of the food under analysis, have gained an increasing relevance over the last years [10].

Among the latter, different analytical methods for determination of volatile compounds combined with multivariate chemometric techniques for VOOs quality testing have been described in the literature and proposed to the industrial sector as fast and high throughput screening techniques [9,11,12,13,14,15,16,17,18].

In particular, as an alternative to headspace gas chromatography-mass spectrometry (HS-GC/MS), which is the most widely used technique to quantify and characterize the profiles of volatile compounds of VOOs thanks to its high sensitivity and selectivity, the application of the HS-GC ion mobility spectrometry (HS-GC-IMS) has been proposed. This technique combines high selectivity and sensitivity with high robustness and cost-efficiency, and has given promising results in discriminating VOOs according to quality grades [9,11,12,14,18] or geographical origin [13,15].

The need to support organoleptic analysis was also reported in a specific call of the Horizon 2020 EU program (H2020-SFS-14a-2014) and is one of the main objectives of the OLEUM project (Horizon 2020, Grant Agreement No. 635690). In the framework of this project, two analytical instrumental techniques, headspace-solid phase micro extraction–gas-chromatography/mass spectrometry (HS-SPME-GC/MS) and flash gas chromatography (FGC) based on the determination of volatile compounds, have been proposed as the most promising rapid screening methods that can support sensory panels in the determination of quality grades.

In a recent work by Quintanilla-Casas and co-authors (2020) [17], the results obtained with HS-SPME-GC/MS with a fingerprinting approach to classify VOO categories has been demonstrated. Herein, a classification model based on minor fraction fingerprints that is able to predict the commercial category of olive oil samples (EVOOs, VOOs, LOOs) obtained by FGC is presented. The FGC is an innovative analytical approach for analysis of volatile compounds of VOOs based on the FGC separation: the headspace of VOOs, previously conditioned, is sampled by a syringe, the volatile organic compounds are adsorbed on a Tenax trap and subsequently desorbed by rapid heating, and, finally, transferred to a FGC step. The elution of analytes runs in parallel using two metal capillary columns with different polarity of the stationary phase. This gives rise to slight differences in the separation capability of molecules that are detected by a flame ionization detector (FID) located at the end of each column.

The main advantage of the FGC technique is its short analysis time (total separation time is 100 s); moreover, its application associated with sensory analysis for calibration and chemometric tools is promising to support the work of panel tests in discriminating samples of different product categories. A classification model, once built, could be easily applied in any laboratory or industry.

The effectiveness of this technique is already demonstrated by previous works aimed to differentiate VOOs according to their geographical origin declared by labels such as “100% Italian” and ‘‘non-100% Italian” oils [19] or “EU” and “extra-EU” [16].

The aim of this study was to classify VOOs according to quality grade, combining FGC data with the multivariate classification technique partial least squares discriminant analysis (PLS-DA). To provide robustness to our model, a set of 331 oils belonging to the three different commercial categories (EVOO, VOO, LOO) involving two harvesting/production years was analyzed. The adopted validation protocol (repeatability and reproducibility tests) and related performance are also shown.

## 2. Materials and Methods

### 2.1. Olive Oil Samples

An initial set of 334 EVOOs, VOOs, and LOOs oils representative of the most common olive cultivars, geographical origin, sensory positive attributes, and sensory defects were sampled. Specifically, in addition to a first set of 180 oils collected during the first year of the OLEUM project (2016–2017 olive season), another set of 154 samples (2017–2018 olive season) was collected and analyzed during the second year (Tables S1–S4 in the Supplementary Materials).

The panel test method was carried out by six panels involved in the OLEUM project as described by Barbieri et al. 2020 [20] and sensory data were expressed as mean of medians. The procedure deals with possible disagreement between panels with a decision tree in order to have definitive classification of samples in which definitive agreement is reached. In agreement with the sensory results reported in Tables S1 and S2 (Supplementary Materials), in the first year of the project 178 of 180 samples were immediately classified by panels (54 EVOO, 78 VOO, and 48 LOO). Classification was not possible for only two samples (UN_10, UP_14), as agreement among panels was not reached on the category (V/L). The sensory evaluation of oils from the second sampling allowed classification of 153 oils (69 EVOO, 51 VOO and 33 LOO); 1 sample was not classified due to an anomalous lemon smell (ZRS_1) and was therefore excluded from the set [20]. For these reasons, the classification model was built on 331 samples.

The oils collected were representative of possible commercial samples and borderline samples that can be the object of disagreement between panels in terms of sensory characteristics. Different aliquots of the samples, stored in the lab at 10–12 °C (for sensory analysis) and at −18 °C (for instrumental analysis), were reconditioned at room temperature before analysis.

### 2.2. Analytical Conditions

The FGC system (FGC-E-nose Heracles II, AlphaMos, Toulouse, France) is based on the technology of ultra-fast gas-chromatography.

The FGC is equipped with two columns working in parallel: a non-polar column (MXT5: 5% diphenyl, 95% methylpolysiloxane, 10 m length and 180 μm diameter) and a polar column (MXT-1701: 14% cyanopropylphenyl/86% dimethyl polysiloxane, 10 m length, 180 μm diameter). At the end of each column, a FID detector is placed and the acquired signal is digitalized every 0.01 s.

The analytical conditions applied were the same described by Melucci et al. 2016 [19]. The only difference was related to the temperature of the conditioning step of the samples before injection: the vial is placed in the auto-sampler (HS 100, CTC Analytics), which moves it in a shaker oven where it remains for 20 min at 40 °C, shaken at 500 rpm.

### 2.3. Validation Protocol

To confirm that the analytical procedure employed has performance capabilities consistent with the required application, a validation strategy for non-targeted approaches was performed.

A QC (quality control) sample, representative of the qualitative and quantitative VOO volatile composition (presence of volatile compounds along the entire interval of the chromatogram), was used. In this study, the QC sample was obtained by pooling the same volume of three case-control samples (1 EVOO, 1 VOO with median of 1.9 for fusty-muddy defect, and 1 VOO with a median of 2.5 for rancid defect) and seven replicates were taken into consideration.

The quality of the instrumental performance intended for fingerprinting analysis was checked by the calculation of the relative standard deviation (RSD) as proposed by the Food and Drug Administration [21]. Specifically, the repeatability (intra-day repeatability and inter-day repeatability performed according to EC 657/2002) [22] of the chromatographic signal evaluated in terms of RSD% of each chromatogram data point, with intensities above noise signal of the replicates of the same QC samples, was considered [23,24].

Prior to RSD calculation, data were aligned using the COW algorithm (correlation optimized warping) [25] and autoscaled (mean-centering followed by division of variable by the standard deviation of that column) to correct shifts in retention time and possible differences in the signal amplification of the instrument. All elaborations were made using PLS Toolbox for Matlab (MatlabR2018a^®^) (Natick, MA, USA).). For calculation of RSD% for each chromatogram data point, the evaluation and exclusion of noise signal is carried out to avoid considering non-relevant RSD%.

For precision, the FDA recommends a RSD not higher than 15% regarding the analytical variability for target analysis, except for concentrations close to the detection limit where a RSD of 20% is acceptable (FDA Bioanalytical Method Validation-Guidance for Industry, 2018). This, in agreement with the trend described by the Horwitz equation for targeted methods [26], demonstrates that the repeatability is strongly correlated with the intensity of the variables.

Although fingerprinting represents a different analytical approach and more variation is expected when doing untargeted analysis, these guidelines are used as a benchmark towards repeatability evaluation. Specifically, for intra-day repeatability, the acceptance criteria were as follows: more than 90% of signals with RSD < 15%; more than 95% of signals with RSD < 20% and distribution of RSD% vs. signal intensity in accordance with the Horwitz equation. For inter-day repeatability or within-lab reproducibility, the acceptance criteria were as follows: more than 85% of signals with RSD < 15%, more than 90% of signals with RSD < 20% and distribution of RSD% vs. signal intensity in accordance with Horwitz’s equation.

In addition, the examination of system performance by checking the signal to noise ratio in standard solutions (instead of the evaluation of representative VOO profiles) to facilitate the assessment and comparison of method sensitivity for other laboratories was proposed. The sensitivity of the analytical system was evaluated by analyzing 2 g of each standard solution in refined olive oil (ethanol 0.05 mg·kg^−1^, CAS Number 64-17-5; assay ≤ 97.2%; density 0.789 g/mL at 25 °C; hexanal, 0.1 mg·kg^−1^ CAS Number 66-25-1; assay ≥ 95% (GC); density 0.815 g/mL at 25 °C; (*E*)-2-hexenal, 0.75 mg·kg^−1^ CAS Number 6728-26-3; assay ≥ 97.0% (GC); density 0.846 g/mL at 25 °C). The S/N (S = intensity of the peak of the compound; N = mean intensity of the noise measured considering the baseline of the chromatographic zone between 43 and 50 s) for the selected analytes in the chromatograms should be >3 (acceptance criteria).

### 2.4. Classification Models

In order to predict the assignment of samples to a specific quality grade, full chromatograms were used to develop classification models. The raw data of each chromatogram, for a total of 19,900 points, were aligned by the COW algorithm and autoscaled using PLS Toolbox for Matlab (MatlabR2018a^®^). Subsequently, the noise was excluded and 8401 points were consecutively selected from first to last peak observed in the chromatogram.

Subsequently, PLS-DA (partial least square discriminant analysis) models [27] were built by using the intensity values of the points as variables X (matrix X), while the commercial categories (EVOO, VOO, LOO) were considered as variable Y. In particular, classification models with 2 categories were developed in sequence: EVOO vs. no-EVOO followed by VOO vs. LOO and LOO vs. no-LOO followed by EVOO vs. VOO, as proposed by Quintanilla-Casas et al. 2020 [17].

The sample dataset was split in calibration (venetian blinds cross validation, including 75% of the samples) and external validation set (25% of the samples) by using the Kennard–Stone method [28]. The dataset was deposited for possible consultation in an on-line repository [29].

The threshold value able to identify the belonging category of each sample into one of the groups was defined by using a probabilistic approach based on Bayes’s rule [30]. Finally, to assess the goodness of the method, the receiver operating characteristic (ROC) curves were evaluated.

## 3. Results and Discussion

### 3.1. Performance of FGC

Most of the procedures proposed in the literature for validation of non-targeted methods focus on post-analytical data treatment and validation of statistical models. Nevertheless, a few studies have investigated control procedures as well as performance criteria and requirements to ensure the consistence of the analytical signal (fingerprint) [24,31].

Conventional performance criteria adopted for targeted methods are not applicable as such to fingerprinting methods. Fingerprinting methods intended for sample classification are not aimed at identification and quantification of analytes, but on finding distinctive patterns that are specific for a given food category (i.e., VOO commercial category) in raw analytical signals (i.e., chromatograms). Therefore, the main constraint of the fingerprinting analytical method is to provide a repeatable and reproducible signal with sufficient sensitivity to collect the information from samples for the final purpose of the method, i.e., quality classification.

For evaluation of intra-day repeatability, the pooled QC sample was analyzed by the same operator with the same equipment and in the same instrument operative conditions within the same day. For each variable (data points), mean value, SD, and RSD% were calculated considering the seven replicates. More than 97.5% of signals presented RSD < 10%, while it achieves 99.8% in correspondence of RSD < 20% (Table 1). To analyze the variability as related to the magnitude of the variables, RSD% was plotted versus signal intensity (data not shown). As expected, data points with RSD > 10% are characterized by low values of intensity. This is in agreement with the trend described by the Horwitz equation for targeted methods [26].

In the case of the inter-day repeatability (within-lab reproducibility), seven replicates of the pooled QC sample were analyzed by the same operator with the same equipment but on different days, consequently involving different environmental conditions, and the mean value, SD, and RSD% were calculated. More than 91% and 99.4% of the signals presented RSD < 10% and RSD < 20%, respectively (Table 1). A relation between intensity and RDS% was also observed in this study, similarly to that previously observed in the intra-day repeatability test.

As the fingerprinting approach intended for sample classification is not aimed in determining the concentration of single analytes, limits of detection or quantification cannot be calculated for the analytical outcome. However, the analytical method needs to be sufficiently sensitive to allow detection of minor constituents to avoid missing any valuable information. 

On this basis, the method’s sensitivity needs to be set as a reference parameter to be evaluated in the validation process. A target-type strategy applied to standard solutions was proposed.

Standard solution compounds were chosen as most representative of the qualitative and quantitative volatile composition of VOOs, especially regarding the presence of volatile compounds over the entire interval of the chromatogram considered in fingerprinting analysis. Differences between the concentrations used for each compound are related to their different amounts generally present in a VOO sample. Results of the S/N are reported in Table 2.

### 3.2. Classification Models

A fingerprinting approach involving chemometric elaboration of the entire profiles in volatile molecules without identification and quantification was applied.

Two different classification strategies were taken into account: (i) a classification model able to discriminate EVOO and no-EVOO samples, followed by a model to classify VOO vs. LOO samples; (ii) a classification model able to discriminate LOO and no-LOO samples, followed by a model to classify VOO vs. EVOO samples.

The results, in terms of percentage and number of correctly classified samples, are reported in Table 3 for cross and external validation, respectively. Regarding the first classification strategy, the percentages of correctly classified samples ranged from 72 to 89% and from 72 to 85%, for cross and external validation, respectively. In particular, the best results were obtained during the second step useful to discriminate VOO vs. LOO. For the second strategy, conceptually more correct in terms of sequence because it first discriminates LOO which are not edible if not refined, the percentage ranged from 78 to 92% and from 73 to 85%, for cross and external validation, respectively. In this case, the highest percentages were reached using the first PLS-DA model (LOO vs. no-LOO). Furthermore, this latter model was the best of all PLS-DA models developed.

In general, the percentages are in the same range as those obtained by other authors who proposed chemometric models to discriminate VOO quality grades according to their volatile profile analyzed by different instrumental techniques [9,17].

**Figure 1:**
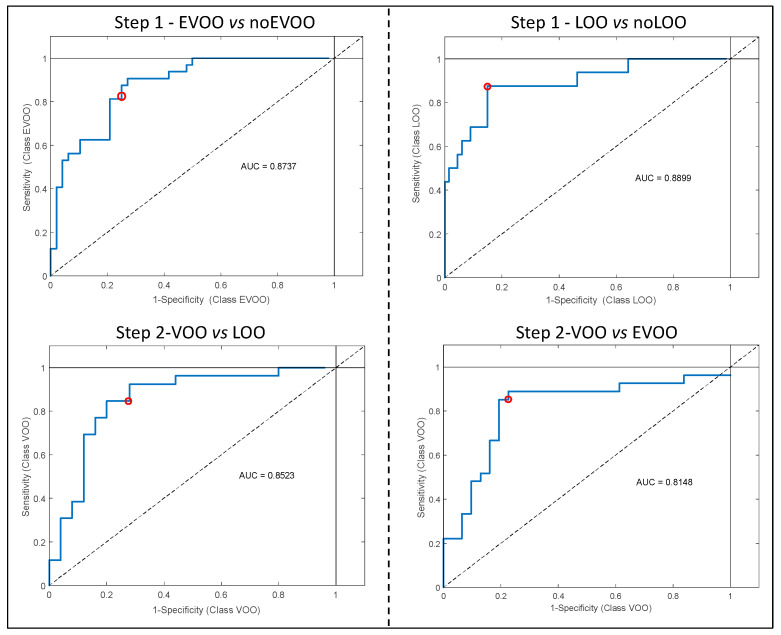
Receiver operating characteristic (ROC) curves of all developed PLS-DA models used to discriminate samples according to quality grade; the red circles identify the sensitivity (number of samples predicted as in the class divided by number actually in the class) and the specificity (number of samples predicted as not in the class divided by actual number not in the class) of the models. EVOO = extra virgin olive oil; VOO = virgin olive oil, LOO = lampante Olive Oil.

The ROC curves (Figure 1) evaluated the sensitivity (number of samples predicted as in the class divided by number actually in the class) and the specificity (number of samples predicted as not in the class divided by actual number not in the class) of all PLS-DA models (external validation) [16]. In particular, the area under the curve (AUC) identifies the degree of discrimination (ranged 0.8148 to 0.8899) and suggests that all the models are characterized by a good degree of discrimination.

**Figure 2:**
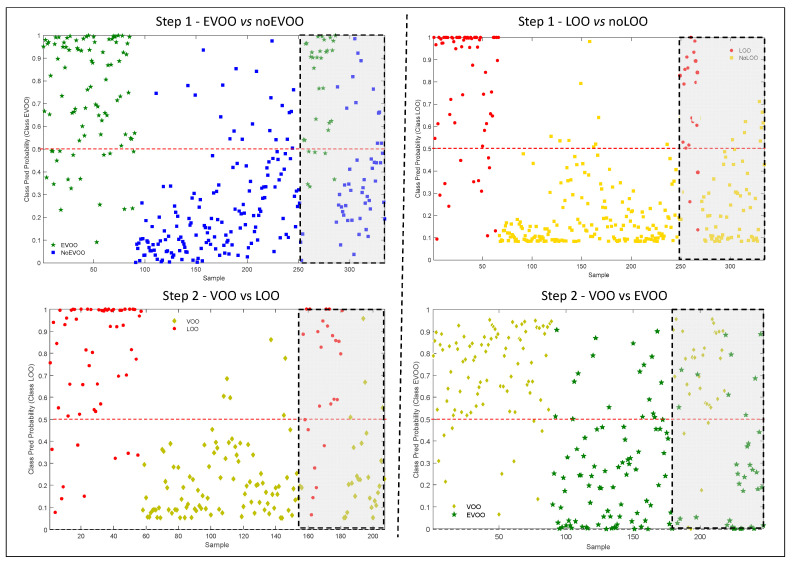
Class prediction probability of all samples used to develop the models, in cross and external validation (grey area). Step 1—EVOO (green star) vs. no-EVOO (blue square); step 2—VOO (yellow diamond) vs. LOO (red circle); step 1—LOO (red circle) vs. no-LOO (yellow square); step 2—VOO (yellow diamond) vs. EVOO (green star). EVOO = extra virgin olive oil; VOO = virgin olive oil, LOO = lampante olive oil.

The results of all the models (cross and external validation), in term of probability of belonging to the correct class, are shown in Figure 2. The threshold value was fixed at 0.5, corresponding to a probability of 50%: a sample classified with a probability lower than this is considered as not correctly grouped [32].

The definition of a probability level, ranging from 50% to 100%, could be a means of identifying uncertain samples that need to be checked by sensory evaluation. In other words, the samples classified with a probability lower than the selected probability level should be submitted to panel test. These procedures would reduce the amount of the samples analyzed by the panel, but at the same time, it would insure the accuracy of the classification.

## 4. Conclusions

Despite the undisputed validity of the panel test, its application is time consuming and expensive. Accordingly, companies and private and public quality control labs could benefit from robust instrumental pre-classifications, which would reduce the number of samples that have to be assessed by panels, or at least prioritize their assessment.

For this reason, the development of rapid screening methods to support the official panel test, to analyze olive oils and differentiate their quality grades, is one of the challenges in the olive oil sector, as reported in the EU framework program Horizon 2020.

In this work, FGC combined with the multivariate statistical technique was applied to discriminate samples according to different quality grades (EVOO, VOO and LOO; examples of GC traces for EVOOs and LOOs are shown in Figure S1 of the Supplementary Materials). The analytical technique proposed herein for fingerprinting olive oils combined with chemometrics was effective in reducing data complexity and time to obtain a response; this rapid screening tool could be adopted for a quick pre-classification of the quality grades, e.g., by control laboratories in companies of the OO sector, before buying or blending EVOOs.

In order to propose a robust chemometric model, a large set of samples (*n* = 331) involving two different harvesting/production years, the most common olive cultivars, geographical origin, sensory positive attributes, and sensory defects, was analyzed. In addition, a validation protocol was adopted for evaluate the reliability of the results.

The proposed analytical fingerprinting method provided repeatable and reproducible signals with sufficient sensitivity to collect valuable information about samples.

FGC associated with the two-category sequential classification model is promising to support sensory analysis in discriminating samples of different product categories. Among the proposed classification strategy, the second (1st step: LOO vs. no-LOO; 2nd step: VOO vs. EVOO) was the best of all PLS-DA models developed with percentages of correctly classified samples ranging from 78 to 92% and from 73 to 85%, for cross and external validation, respectively.

This analytical approach is very fast, and, in fact, only around 200 s are needed to analyze a single sample. The classification model, built by using a high number of robust samples classified by sensorial analysis and representative of the commercial variability (here we used a decision tree and six panels to ensure their classification) is easily applicable in any laboratory or industry.

Future studies could be addressed to the implementation of this methodology, even in relation to an increasing interest of the food sector towards volatile compounds and more widespread use of instruments such as FGC, which are less common in quality control laboratories. An even wider sampling phase including other variables among oils, since they are natural products, could lead to a better control of classifications and would lead to implementation of this technique to a broader extent. Lastly, the use of other statistical approaches, such as nonlinear techniques, could be investigated in order to improve the results of classification.

## Figures and Tables

**Table 1:** Frequency of each relative standard deviation percentage (RSD%) class obtained for intra-day and inter-day repeatability evaluated on the quality control (QC) sample.

Intra-Day Repeatability			Inter-Day Repeatability		
Class (RSD%)	Frequency	%	Class (RSD%)	Frequency	%
10	8185	97.5	10	7690	91.5
15	166	1.5	15	552	6.6
20	33	0.8	20	106	1.3
30	17	0.2	30	16	0.2
40	0	0	40	37	0.4
50	0	0	50	0	0
60	0	0	60	0	0
70	0	0	70	0	0
80	0	0	80	0	0
90	0	0	90	0	0
100	0	0	100	0	0

**Table 2:** Concentration (mg·kg^−1^) of each compound included in the standard solution used for method’s sensitivity evaluation and related S/N. The standard mix were prepared by spiking refined olive oil with each compound and analysed by flash gas chromatography (FGC). S = intensity of the peak of the compound; N = mean intensity of the noise measured considering the baseline of the chromatographic zone between 43 and 50 s.

Compound	Retention Time	Concentration (mg·kg^−1^)	S/N
Ethanol	21.8	0.05	3.84 ± 0.99
Hexanal	55.6	0.1	5.55 ± 0.96
(*E*)-2-Hexenal	62.0	0.75	4.42 ± 1.82

**Table 3:** Results in terms of percentage and number of samples correctly classified in cross and external validation of the two classification strategies applied based on the partial least squares-discriminant analysis (PLS-DA) sequential model. EVOO = extra virgin olive oil; VOO = virgin olive oil, LOO = lampante olive oil.

**1st CLASSIFICATION STRATEGY**		**2nd CLASSIFICATION STRATEGY**	
**1st Step: EVOO vs. no-EVOO**		**1st Step: LOO vs. no-LOO**	
Cross validation	External validation	Cross validation	External validation
EVOO: 70/90 (78%)	EVOO: 26/32 (81%)	LOO: 50/61 (81%)	LOO: 17/20 (85%)
No-EVOO: 132/164 (81%)	No-EVOO: 37/48 (77%)	No-LOO: 172/188 (92%)	No-LOO: 55/65 (85%)
TOTAL: 202/254 = 80%	TOTAL: 63/80 = 79%	TOTAL: 222/249 = 89%	TOTAL: 72/85 = 85%
**2nd Step: VOO vs. LOO**		**2nd Step: VOO vs. EVOO**	
Cross validation	External validation	Cross validation	External validation
VOO: 88/99 (89%)	VOO: 22/26 (85%)	VOO: 84/95 (88%)	VOO: 23/27 (85%)
LOO: 41/57 (72%)	LOO: 18/25 (72%)	EVOO: 74/94 (78%)	EVOO: 24/33 (73%)
TOTAL: 129/156 = 83%	TOTAL: 41/51 = 78%	TOTAL: 158/189 = 84%	TOTAL: 47/60 = 78%

## Supplementary Materials

The following are available online at https://www.mdpi.com/2304-8158/9/7/862/s1, Table S1: Sensory results of samples from the first year. Table S2: Sensory results of samples from the second year. Table S3: available information on samples collected and evaluated during the first year of the Oleum project. Table S4: available information on samples collected and evaluated during the second year of the Oleum project. Figure S1: overlapping of the GC traces of extra virgin (EVOO) and lampante (LOO) samples.
