# Supplementary material for: Flash Gas Chromatography in Tandem with Chemometrics: A Rapid Screening Tool for Quality Grades of Virgin Olive Oils
Source: Foods. 2020 Jul 2;9(7):862. doi: 10.3390/foods9070862 (PMC7404474; doi:10.3390/foods9070862)

Table S1. Set of 180 samples collected and evaluated during the first year of the Oleum project with their codes and quality grades established by sensory evaluation. EVOO: Extra Virgin olive oil; VOO: virgin olive oil; LOO: Lampante olive oil; nc: not classified.

Code	Quality grade	Code	Quality grade	Code	Quality grade
EU_1	**EVOO**	EU_21	**VOO**	UP_8	**VOO**
EU_11	**EVOO**	EU_22	**VOO**	UZ_1	**VOO**
EU_13	**EVOO**	EU_23	**VOO**	UZ_10	**VOO**
EU_14	**EVOO**	EU_26	**VOO**	UZ_11	**VOO**
EU_20	**EVOO**	EU_27	**VOO**	UZ_13	**VOO**
EU_3	**EVOO**	EU_28	**VOO**	UZ_15	**VOO**
EU_30	**EVOO**	EU_29	**VOO**	UZ_16	**VOO**
IP_1	**EVOO**	EU_31	**VOO**	UZ_20	**VOO**
IP_10	**EVOO**	EU_32	**VOO**	UZ_26	**VOO**
IP_11	**EVOO**	EU_33	**VOO**	UZ_3	**VOO**
IP_2	**EVOO**	EU_34	**VOO**	EU_10	**LOO**
IP_3	**EVOO**	EU_35	**VOO**	EU_18	**LOO**
IP_4	**EVOO**	EU_36	**VOO**	EU_24	**LOO**
IP_5	**EVOO**	EU_4	**VOO**	EU_25	**LOO**
IP_6	**EVOO**	EU_5	**VOO**	EU_6	**LOO**
IP_7	**EVOO**	EU_8	**VOO**	EU_7	**LOO**
IP_8	**EVOO**	IP_14	**VOO**	EU_9	**LOO**
IP_9	**EVOO**	IP_15	**VOO**	IP_12	**LOO**
IT _2	**EVOO**	IP_17	**VOO**	IP_13	**LOO**
IT_3	**EVOO**	IP_18	**VOO**	IP_16	**LOO**
IT_5	**EVOO**	IP_20	**VOO**	IP_19	**LOO**
IT_6	**EVOO**	IP_21	**VOO**	IP_22	**LOO**
IT_7	**EVOO**	IP_23	**VOO**	IP_24	**LOO**
IT_9	**EVOO**	IP_31	**VOO**	IP_25	**LOO**
UN_11	**EVOO**	IT_10	**VOO**	IP_26	**LOO**
UN_12	**EVOO**	IT_11	**VOO**	IP_27	**LOO**
UN_19	**EVOO**	IT_4	**VOO**	IP_28	**LOO**
UN_2	**EVOO**	IT_8	**VOO**	IP_29	**LOO**
UN_24	**EVOO**	UN_1	**VOO**	IP_30	**LOO**
UN_28	**EVOO**	UN_13	**VOO**	IP_32	**LOO**
UN_29	**EVOO**	UN_18	**VOO**	IT_1	**LOO**
UN_3	**EVOO**	UN_20	**VOO**	IT_12	**LOO**
UN_30	**EVOO**	UN_21	**VOO**	IT_13	**LOO**
UN_34	**EVOO**	UN_23	**VOO**	IT_14	**LOO**
UN_4	**EVOO**	UN_25	**VOO**	UN_14	**LOO**
UN_41	**EVOO**	UN_26	**VOO**	UN_15	**LOO**
UN_5	**EVOO**	UN_27	**VOO**	UN_16	**LOO**
UN_8	**EVOO**	UN_31	**VOO**	UN_17	**LOO**

UP_11	**EVOO**	UN_32	**VOO**	UN_22	**LOO**
UP_17	**EVOO**	UN_33	**VOO**	UN_35	**LOO**
UP_18	**EVOO**	UN_36	**VOO**	UN_37	**LOO**
UP_2	**EVOO**	UN_38	**VOO**	UP_10	**LOO**
UP_21	**EVOO**	UN_39	**VOO**	UP_20	**LOO**
UP_22	**EVOO**	UN_40	**VOO**	UP_26	**LOO**
UP_24	**EVOO**	UN_6	**VOO**	UP_5	**LOO**
UP_27	**EVOO**	UN_7	**VOO**	UP_9	**LOO**
UP_3	**EVOO**	UN_9	**VOO**	UZ_27	**LOO**
UP_4	**EVOO**	UP_1	**VOO**	UZ_12	**LOO**
UZ_18	**EVOO**	UP_12	**VOO**	UZ_14	**LOO**
UZ_2	**EVOO**	UP_13	**VOO**	UZ_17	**LOO**
UZ_23	**EVOO**	UP_15	**VOO**	UZ_19	**LOO**
UZ_24	**EVOO**	UP_16	**VOO**	UZ_21	**LOO**
UZ_8	**EVOO**	UP_19	**VOO**	UZ_22	**LOO**
UZ_9	**EVOO**	UP_23	**VOO**	UZ_25	**LOO**
EU_12	**VOO**	UP_25	**VOO**	UZ_4	**LOO**
EU_15	**VOO**	UP_28	**VOO**	UZ_5	**LOO**
EU_16	**VOO**	UP_29	**VOO**	UZ_6	**LOO**
EU_17	**VOO**	UP_30	**VOO**	UZ_7	**LOO**
EU_19	**VOO**	UP_6	**VOO**	UN_10	nc
EU_2	**VOO**	UP_7	**VOO**	UP_14	nc

Table S2. Set of 154 samples collected and evaluated during the second year of the Oleum project with their codes and quality grades established by sensory panels. EVOO: Extra Virgin olive oil; VOO: virgin olive oil; LOO: Lampante olive oil.

Code	Quality grade	Code	Quality grade	Code	Quality grade
EU_59	**VOO**	IT_17	**EVOO**	UZ_30	**LOO**
EU_63	**EVOO**	IT_19	**EVOO**	UZ_31	**LOO**
EU_64	**VOO**	IT_22	**VOO**	UZ_37	**LOO**
EU_65	**VOO**	IT_29	**VOO**	UZ_45	**LOO**
EU_72	**VOO**	IT_32	**EVOO**	UZ_50	**LOO**
EU_76	**EVOO**	UN_46	**EVOO**	ZRS_2	**EVOO**
IP_33	**EVOO**	UN_54	**VOO**	ZRS_3	**EVOO**
IP_41	**EVOO**	UN_59	**VOO**	ZRS_7	**VOO**
IP_42	**VOO**	UN_61	**LOO**	ZRS_8	**VOO**
IP_45	**VOO**	UN_67	**EVOO**	ZRS_9	**EVOO**
IP_55	**LOO**	UN_68	**EVOO**	ZRS_25	**EVOO**
IP_57	**VOO**	UZ_34	**EVOO**	EU_71	**EVOO**
IT_15	**VOO**	UZ_35	**LOO**	EU_73	**EVOO**
IT_20	**VOO**	UZ_38	**LOO**	EU_75	**LOO**
IT_27	**LOO**	UZ_39	**EVOO**	IP_37	**EVOO**

IT_34	**EVOO**	UZ_40	**LOO**	IP_40	**EVOO**
IT_35	**VOO**	UZ_42	**LOO**	IP_44	**VOO**
IT_38	**VOO**	UZ_44	**VOO**	IP_50	**VOO**
IT_41	**EVOO**	ZRS_4	**EVOO**	IP_53	**LOO**
UN_42	**EVOO**	ZRS_6	**EVOO**	IT_21	**VOO**
UN_43	**VOO**	ZRS_13	**EVOO**	IT_23	**EVOO**
UN_44	**VOO**	ZRS_14	**EVOO**	IT_25	**VOO**
UN_55	**VOO**	ZRS_17	**VOO**	IT_26	**LOO**
UN_56	**EVOO**	ZRS_21	**LOO**	IT_30	**VOO**
UN_57	**VOO**	ZRS_24	**EVOO**	IT_33	**EVOO**
UN_60	**VOO**	EU_57	**VOO**	IT_37	**EVOO**
UZ_28	**EVOO**	EU_61	**EVOO**	IT_39	**VOO**
UZ_33	**VOO**	EU_66	**EVOO**	UN_48	**EVOO**
UZ_36	**EVOO**	EU_67	**EVOO**	UN_50	**EVOO**
UZ_46	**VOO**	IP_35	**EVOO**	UN_51	**VOO**
UZ_47	**LOO**	IP_36	**EVOO**	UN_52	**EVOO**
UZ_52	**EVOO**	IP_39	**EVOO**	UN_53	**VOO**
ZRS_1	Anomalous	IP_43	**EVOO**	UN_58	**EVOO**
ZRS_5	**EVOO**	IP_47	**LOO**	UN_64	**LOO**
ZRS_16	**EVOO**	IP_54	**LOO**	UN_65	**EVOO**
ZRS_20	**LOO**	IT_16	**LOO**	UN_103	**EVOO**
ZRS_22	**LOO**	IT_18	**EVOO**	UZ_32	**VOO**
ZRS_23	**EVOO**	IT_24	**EVOO**	UZ_41	**LOO**
EU_55	**VOO**	IT_28	**LOO**	UZ_43	**VOO**
EU_56	**EVOO**	IT_31	**EVOO**	UZ_48	**VOO**
EU_60	**VOO**	IT_36	**EVOO**	UZ_49	**VOO**
EU_68	**EVOO**	IT_40	**VOO**	UZ_51	**LOO**
EU_69	**EVOO**	IT_42	**VOO**	ZRS_10	**VOO**
EU_70	**VOO**	UN_45	**EVOO**	ZRS_11	**VOO**
EU_74	**EVOO**	UN_47	**EVOO**	ZRS_12	**VOO**
EU_77	**EVOO**	UN_49	**VOO**	ZRS_15	**EVOO**
IP_34	**EVOO**	UN_62	**LOO**	ZRS_18	**EVOO**
IP_38	**EVOO**	UN_63	**LOO**	ZRS_19	**VOO**
IP_46	**LOO**	UN_66	**EVOO**		
IP_51	**EVOO**	UN_69	**EVOO**		
IP_52	**LOO**	UN_104	**VOO**		
IP_56	**LOO**	UZ_29	**LOO**		

Table S3. Set of 180 samples collected and evaluated during the first year of the Oleum project with available information on geographical origin of the olives, olive typology (olive variety/varieties, PDO, PGI), mill location, processing parameters.

Sample code	Geographical origin of the olives	Olive typology (olive variety/varieties, PDO, PGI)	Mill location	Technology parameters (press/2-phases/3-phases, capacity of the production line)	Technology parameters (filtered, not filtered)
EU_1	Italy	Coratina	Apulia	Unknown	Unknown
EU_2	Italy	Coratina	Unknown	Unknown	Unknown
EU_3	Italy	Frantoio	Calabria	Unknown	Unknown
EU_4	Italy	Castiligionese	Abruzzo	Unknown	Unknown
EU_5	Italy	Unknown	Apulia	Unknown	Unknown
EU_6	Spain	Arbequina	Cambrils	Unknown	Unknown
EU_7	Spain	Arbequina	Cambrils	Unknown	Unknown
EU_8	Italy	Leccino, Frantoio, Moraiolo	Tuscany	Unknown	Unknown
EU_9	Italy	Leccino, Frantoio, Moraiolo	Tuscany	Unknown	Unknown
EU_10	Italy	Leccino, Frantoio, Pendolino	Tuscany	Unknown	Unknown
EU_11	Italy	Leccino, Frantoio, Pendolino	Tuscany	Unknown	Unknown
EU_12	Italy	Leccino, Frantoio, Pendolino	Tuscany	Unknown	Unknown
EU_13	Italy	Arbequina	Tuscany	Unknown	Unknown
EU_14	Spain	Unknown	Sevilla	Unknown	Unknown
EU_15	Spain	Unknown	Sevilla	Unknown	Unknown
EU_16	Spain	Unknown	Andalusia	Unknown	Unknown
EU_17	Spain	Unknown	Andalusia	Unknown	Unknown

EU_18	Spain	Unknown	Cordoba	Unknown	Unknown
EU_19	Spain	Unknown	Unknown	Unknown	Unknown
EU_20	Spain	Unknown	Unknown	Unknown	Unknown
EU_21	Spain	Unknown	Unknown	Unknown	Unknown
EU_22	Spain	Unknown	Unknown	Unknown	Unknown
EU_23	Spain	Unknown	Unknown	Unknown	Unknown
EU_24	Spain	Unknown	Unknown	Unknown	Unknown
EU_25	Spain	Unknown	Subbetica	Unknown	Unknown
EU_26	Spain	Unknown	La Roda	Unknown	Unknown
EU_27	Spain	Unknown	Posadas	Unknown	Unknown
EU_28	Spain	Unknown	La Roda	Unknown	Unknown
EU_29	Spain	Unknown	Unknown	Unknown	Unknown
EU_30	Spain	Unknown	Unknown	Unknown	Unknown
EU_31	Spain	Unknown	Unknown	Unknown	Unknown
EU_32	Spain	Unknown	Unknown	Unknown	Unknown
EU_33	Spain	Unknown	Unknown	Unknown	Unknown
EU_34	Spain	Unknown	Unknown	Unknown	Unknown
EU_35	Spain	Unknown	Unknown	Unknown	Unknown
EU_36	Spain	Unknown	Unknown	Unknown	Unknown
IP_1	Croatia	blend of Leccino (90%) and Pendolino (10%)	Novigrad (Istria)	3-phases, Alfa-laval, 1500 kg/hour capacity	not filtered, naturally sedimented and decanted

IP_2	Croatia	blend of Picholene (55%) and Lecio del Corno (45%)	Novigrad (Istria)	3-phases, Alfa-laval, 1500 kg/hour capacity	not filtered, naturally sedimented and decanted
IP_3	Croatia	blend of Ascolana tenera (50%), Itrana (30%), Frantoio (20%)	Novigrad (Istria)	3-phases, Alfa-laval, 1500 kg/hour capacity	not filtered, naturally sedimented and decanted
IP_4	Croatia	blend of Buža puntoža (40%), Rosinjola (30%) and Bova (30%)	Novigrad (Istria)	3-phases, Alfa-laval, 1500 kg/hour capacity	not filtered, naturally sedimented and decanted
IP_5	Croatia	Istarska bjelica (100%)	Novigrad (Istria)	3-phases, Alfa-laval, 1500 kg/hour capacity	not filtered, naturally sedimented and decanted
IP_6	Croatia	blend of Ascolana tenera- Itrana-Frantoio	Novigrad (Istria)	3-phases, Alfa-laval, 1500 kg/hour capacity	not filtered, naturally sedimented and decanted
IP_7	Croatia	Buža puntoža (100%)	Umag (Istria)	2-phases, 500 kg/hour capacity	not filtered, naturally sedimented and decanted
IP_8	Croatia	Buža puntoža (100%)	Umag (Istria)	2-phases, 500 kg/hour capacity	not filtered, naturally sedimented and decanted
IP_9	Croatia	Picholene (100%)	Novigrad (Istria)	3-phases, Alfa-laval, 1500 kg/hur capacity	not filtered, naturally sedimented and decanted
IP_10	Croatia	blend of Plominka and Šimjaca - (PDO- Extra virgin olive oil -Cres)	Island Cres, Croatia	2-phases, 1200 kg/hour	not filtered
IP_11	Croatia	Oblica (100%)	Postira, island Brač, Croatia	2-phases, 1200 kg/hour	not filtered
IP_12	Croatia	Oblica (100%)	Milna, island Brač, Croatia	3-phases, 1200 kg/hour capacity	not filtered
IP_13	Croatia	Oblica (90%)	Island Brač, Croatia	2-phases, 2500 kg/hour	not filtered; naturally sedimented and decanted
IP_14	Croatia	blend of Leccino (70%) and Buža (30%)	Vodnjan (Istria), Croatia	2-phases, 3500 kg/hour	not filtered
IP_15	Croatia	Oblica (100%)	Primošten Burni, Croatia	2-phases, 2500 kg/hour	filtered
IP_16	Croatia	Oblica (100%)	Island Brač, Croatia	press, 400 kg/h	not filtered; naturally sedimented and decanted
IP_17	Croatia	Oblica (100%)	Murter, Croatia	2-phases, 2000 kg/hour	filtered
IP_18	Greece	Koroneiki (100%)	Gargaliani, Greece,	2-phases, 10 tons/hour	not filtered

IP_19	Spain	blend of Hojiblanco-Pajarero	Llanos del Espinar, Cordoba	2-phases	not filtered; naturally sedimented and decanted
IP_20	Italy	Sinopolese (PGI olio di Calabria)	Seminara (RC), Calabria	2-phases and half (Alfa laval)	not filtered
IP_21	Italy	Ottobratica (PGI olio di Calabria)	Seminara (RC), Calabria	2-phases and half (Alfa laval)	not filtered
IP_22	Spain	Hojiblanco	Cordoba	2-phases	not filtered
IP_23	Italy	Ogliarola	Brindisi, Apulia	2-phases	not filtered
IP_24	Croatia	Blend of Oblica (90%) and Levantinka (10%)	Island Brač, Croatia	2-phases, 2500 kg/hour	not filtered; naturally sedimented and decanted
IP_25	Croatia	Oblica (100%)	Island Brač, Croatia	press, 400 kg/h	not filtered; naturally sedimented and decanted
IP_26	Croatia	blend of Leccino (50%), Coratina, Ascolana tenera, Picholene (50%)	Island Pašman, Croatia	2-phases, 500 kg/hour	not filtered
IP_27	Croatia	blend of Leccino and Pendolino	Kaštel Stari, Croatia	press, 300 kg/h	not filtered
IP_28	Croatia	Leccino (100%)	Novigrad (Istria)	3-phases, Alfa-laval, 1500 kg/hour capacity, processing temperature 25 °C	not filtered, sedimentation for about 1 month and decantation
IP_29	Croatia	Blend of autochtonous varieties (40% Rosinjola, 40% Buža Puntoža, 20% Istarska bjelica)	Novigrad (Istria)	3-phases, Alfa-laval, 1500 kg/hour capacity, processing temperature 25 °C	not filtered, sedimentation for about 1 month and decantation
IP_30	Croatia	Istarska bjelica (100%)	Novigrad (Istria)	3-phases, Alfa-laval, 1500 kg/hour capacity, processing temperature 25 °C	not filtered, sedimentation for about 1 month and decantation
IP_31	Spain	blend of Hojiblanco-Pajarero-Picual	Llanos del Espinar, Cordoba	2-phases	not filtered; decanted
IP_32	Croatia	Oblica (100%)	Postira, island Brač, Croatia	Hydraulic press, 300 kg capacity	not filtered
IT_1	France	Oliviere	Hérault	2-phases	non filtered
IT_2	Spain	P.D.O. Estepa	Spain	2-phases	filtered
IT_3	Italy	Coratina + Ogliarola	Apulia	3-phases	not filtered

IT_4	Spain	Unknown	Spain	2-phases	not filtered
IT_5	Spain	Hojiblanca	Almeria	2-phases	filtered
IT_6	Spain	Hrbequina	Andalusia	2-phases	filtered
IT_7	Spain	Picual	Andalusia	2-phases	filtered
IT_8	Tunisia	Chetoui + Chemlali	Sfax zone	2-phases	not filtered
IT_9	Spain	Arbequina + Hojiblanca	Spain	2-phases	not filtered
IT_10	Spain	Arbequina + Hojiblanca	Spain	2-phases	not filtered
IT_11	Spain	Unknown	Spain	2-phases	not filtered
IT_12	Spain	Arbequina	Spain	2-phases	not filtered
IT_13	Morocco	Arbequina	Morocco	Unknown	not filtered
IT_14	Spain	Unknown	Spain	2-phases	not filtered
UN_1	Spain	Unknown	Spain	Unknown	Unknown
UN_2	Italy	Nocellara del Belice	Sicily	2 phases and 3 phases	filtered
UN_3	Italy	Biancolilla	Sicily	3 phases	filtered
UN_4	Italy	Coratina	Apulia	3 phases	filtered
UN_5	Italy	Nocellara del Belice	Sicily	2 phases and 3 phases	filtered
UN_6	UE	Arbequina	Saragozza	3 phase	filtered
UN_7	Italy	Coratina	Apulia	3 phases	filtered
UN_8	Italy	Leccino, Frantoio, Moraiolo as reported on Tuscany PGI	Florence, Tuscany	3 phases	not filtered
UN_9	Spain	Picual	Cordoba	900 tons olive/day	filtered
UN_10	Greece	Koroneiki	Region of Trifilia Messinia	2 phases	not filtered

UN_11	Italy	Coratina	Andria	2 phases	not filtered
UN_12	Italy	Coratina	Andria	Pieralisi, 2 phases, 90.000-120.000 l/day	not filtered
UN_13	Spain	Arbequina	Albacete	2 phases, 250 tons/day	not filtered
UN_14	Italy	Frantoio-Moraiolo	Unknown	Unknown	Unknown
UN_15	Greece	Koroneki	Unknown	Unknown	Unknown
UN_16	Greece	Koroneki	Unknown	Unknown	Unknown
UN_17	Italy	Unknown	Unknown	Unknown	Unknown
UN_18	Greece	Koroneki	Ilias region	2 mills: 1) 2 phases; 2) 3 phases	not filtered
UN_19	Spain	prevalence of Manzanilla	Sevilla	2 phases	not filtered
UN_20	Spain	Picual + Hojiblanco	Lucena, Cordoba	2 phases	not filtered
UN_21	Italy	Coratina	Foggia	2 phases, 15 tons/day	not filtered
UN_22	Morocco	Unknown	Meknes	2- phases (400 tons/day)	not filtered
UN_23	Morocco	Picholine marocaine	Meknes	2- phases (400 tons/day)	not filtered
UN_24	Morocco	Arbequina	Meknes	2- phases (400 tons/day)	not filtered
UN_25	Morocco	Arbequina	Meknes	2- phases (400 tons/day)	not filtered
UN_26	Argentina	Arbequina	Mendoza	2- phases	not filtered
UN_27	Argentina	Arbequina organic	Mendoza	2- phases	not filtered
UN_28	Italy	Coratina	Unknown	Unknown	filtered
UN_29	Spain	Manzanilla	Unknown	Unknown	not filtered
UN_30	Italy	Nostrana di Brisighella P.D.O. Brisighella	Brisighella, Emilia-Romagna	3 phases (Alfa Laval) 15 q.li/h capacity	partially filtered
UN_31	Greece	Koroneiki organi	Kalamata, Messinia	2 phases	not filtered

UN_32	Greece	Koroneiki organic	Iraklio, Creta	2 phases	not filtered
UN_33	Greece	Koroneiki, organic, P.D.O. Kolymvari Kritis	Messará, Creta	2 phases	not filtered
UN_34	Greece	Koroneiki	Olimpia	2 phases	not filtered
UN_35	Greece	Manaki	Nafplio	2 phases	not filtered
UN_36	Greece	Megaritiki	Astros, Argolide	2 phases	not filtered
UN_37	Spain	Unknown	Antequera (Málaga)	2 phases	not filtered
UN_38	Spain	Hojiblanca (100, %)	Antequera (Málaga)	2 phases	not filtered
UN_39	Spain	Others (62.6%) Hojiblanca (37.4%)	El Burgo (Málaga) (62.6%); Villanueva del Rosario (Málaga) (37.4%)	2 phases	not filtered
UN_40	Spain	Unknown	Antequera, Malaga	2 phases	not filtered
UN_41	Chile	Unknown	Valle de Colchagua, Chile	2 phases	not filtered
UP_1	Slovenia	blend of Istrska belica , Leccino and other	Slovenia	2-phases	filtered
UP_2	Slovenia	Istrska belica	Slovenia	press	filtered
UP_3	Slovenia	blend of Istrska belica , Leccino and other	Slovenia	2-phases	filtered
UP_4	Slovenia	blend of Istrska belica , Leccino and other	Slovenia	2-phases	filtered
UP_5	Slovenia	blend of Istrska belica , Leccino and other	Slovenia	3-phases	filtered
UP_6	EU Blend	Unknown	Unknown	Unknown	filtered
UP_7	MEDIT Blend	Unknown	Unknown	Unknown	filtered
UP_8	Greece	Kalamata Selia	Greece	Unknown	filtered
UP_9	Greece	Lyrakis	Greece	Unknown	filtered
UP_10	MEDIT Blend	Unknown	Unknown	Unknown	filtered
UP_11	Slovenia	blend of Istrska belica , Leccino and other	Slovenia	2-phases	filtered

UP_12	Slovenia	blend of Istrska belica , Leccino and other	Slovenia	press	unfiltered
UP_13	slovenia	Istrska belica	Slovenia	press	unfiltered
UP_14	EU Blend	Unknown	Unknown	Unknown	filtered
UP_15	Slovenia	blend of Istrska belica , Leccino and other	Slovenia	2-phases	filtered
UP_16	Slovenia	blend of Istrska belica , Leccino and other	Slovenia	2-phases	unfiltered
UP_17	Slovenia	blend of Istrska belica , Leccino and other	Slovenia	2-phases	filtered
UP_18	Slovenia	blend of Istrska belica , Leccino and other	Slovenia	2-phases	filtered
UP_19	EU Blend	Unknown	Unknown	Unknown	filtered
UP_20	Slovenia	blend of Istrska belica , Leccino and other	Slovenia	3-phases	filtered
UP_21	Slovenia	Istrska belica, Leccino, Maurino	Slovenia	3-phases	filtered
UP_22	SLovenia	Istrska belica, Leccino, Maurino	Slovenia	2-phases	filtered
UP_23	EU Blend	Unknown	Unknown	Unknown	filtered
UP_24	Slovenia	PDO Slovenska Istra; blend Istrska belica, Leccino, Maurino	Slovenia	2-phases	filtered
UP_25	Croatia	Buga, Leccino	Croatia	3-phases	filtered
UP_26	EU Blend	Unknown	Unknown	Unknown	unfiltered
UP_27	Slovenia	PDO Slovenska Istra, Istrska belica	Slovenia	2-phases	filtered
UP_28	EU Blend	Unknown	Unknown	Uknown	filtered
UP_29	EU Blend	Unknown	Unknown	Uknown	filtered
UP_30	Tunisia	Unknown	Tunisia	Uknown	filtered
UZ_1	Turkey	Ayvalik Variety	Edremit-North Eagean	2-phases	filtered
UZ_2	Turkey	Ayvalik+Domat blend	Akhisar-Manisa Egean	3-phases	non filtered

UZ_3	Turkey	Memecik	Karpuzlu-Aydın South Egean	3-phases	non filtered
UZ_4	Turkey	Memecik	Söke-Aydın South Egean	3-phases	non filtered
UZ_5	Turkey	Memecik	Ortaklar-Aydın South Egean	3-phases	non filtered
UZ_6	Turkey	Memecik	Oren-Milas Muğla South Egean	3-phases	non filtered
UZ_7	Turkey	Memecik	Milas Muğla South Egean	2-phases	non filtered
UZ_8	Turkey	Memecik + Gemlik blend	Aydın Sultanhisar	3-phases	non filtered
UZ_9	Turkey	Memecik	Aydın Germencik	3-phases	non filtered
UZ_10	Turkey	Memecik	Aydın Sultanhisar	3-phases	non filtered
UZ_11	Turkey	Memecik	Aydın Çine	3-phases	non filtered
UZ_12	Turkey	Memecik	Aydın Çine	3-phases	non filtered
UZ_13	Turkey	Ayvalik	Ayvalik Gömeç North Egean	2-phases	non filtered
UZ_14	Turkey	Ayvalik	Ayvalik Gömeç North Egean	3-phases	non filtered
UZ_15	Turkey	Ayvalik	Karaağaç Burhaniye North Egean	3-phases	non filtered
UZ_16	Turkey	Ayvalik	Karaağaç Burhaniye North Egean	3-phases	non filtered
UZ_17	Turkey	Edremit	Edremit	3-phases	non filtered
UZ_18	Turkey	Ayvalik	Ayvalik Gömeç North Egean	2-phases	non filtered
UZ_19	Turkey	Ayvalik	Ayvalik	3-phases	non filtered
UZ_20	Turkey	Ayvalik	Edremit-North Eagean	3-phases	non filtered
UZ_21	Turkey	Memecik	Kemalpaşa İzmir	2-phases	non filtered
UZ_22	Turkey	İzmir yağlık	Kemalpaşa İzmir	2-phases	non filtered
UZ_23	Turkey	Domat	Kemalpaşa İzmir	2-phases	non filtered

UZ_24	Turkey	Memecik	Kemalpaşa İzmir	2-phases	non filtered
UZ_25	Turkey	Karamani, Haşebi, Gemlik blended	Altınözü-Antakya	3-phases	non filtered
UZ_26	Turkey	Karamani, Haşebi blended	Altınözü-Antakya	3-phases	non filtered
UZ_27	Turkey	Karamani, Haşebi blended	Altınözü-Antakya	3-phases	non filtered

Table S4. Set of 154 samples collected and evaluated during the second year of the Oleum project with available information on geographical origin of the olives, olive typology (olive variety/varieties, PDO, PGI), mill location, processing parameters.

Sample code	Geographical origin of the olives	Olive typology (olive variety/varieties, PDO, PGI)	Mill location	Technology parameters (press/2-phases/3-phases, capacity of the production line)	Technology parameters (filtered, not filtered)
EU_55	Portugal	Arbequina	Beja	Unknown	Unknown
EU_56	Portugal	Arbequina	Beja	Unknown	Unknown
EU_57	Spain	Unknown	Andalusia	Unknown	Unknown
EU_59	Spain	Unknown	Jaen	Unknown	Unknown
EU_60	Spain	Manzanilla, Hojiblanca, Picual	Sevilla	Unknown	Unknown
EU_61	Spain	Manzanilla, Hojiblanca, Picual	Sevilla	Unknown	Unknown
EU_63	Spain	Unknown	Toledo	Unknown	Unknown
EU_64	Spain	Unknown	Toledo	Unknown	Unknown
EU_65	Spain	Unknown	Toledo	Unknown	Unknown
EU_66	Spain	Unknown	Toledo	Unknown	Unknown
EU_67	Greece	Koroneiki	Messenia	Unknown	Unknown
EU_68	Italy	Carolea	Calabria	Unknown	Unknown
EU_69	Greece	Psioelies	Crete	Unknown	Unknown
EU_70	Greece	Manaki	Peloponnes	Unknown	Unknown
EU_71	Italy	Dritta (80%), Leccino (20%)	Abruzzo	Unknown	Unknown
EU_72	Tunisia	Unknown	Unknown	Unknown	Unknown
EU_73	Italy	Frantoio	Foligno, Umbria	3-phases	filtered

EU_74	Italy	Peranzana	Bari, Apulia	2-phases	not filtered
EU_75	Italy	Ottobratica	Reggio Calabria, Calabria	3-phases	not filtered
EU_76	Italy	Leccino	Lecce, Apulia	2-phases	not filtered
EU_77	Italy	Cellina di Nardò	Lecce, Apulia	2-phases	not filtered
IP_33	Croatia	Picholene	Novigrad (Istria), Croatia	3-phases, Alfa-laval, 1500 kg/hour capacity	not filtered
IP_34	Croatia	Lecco del Corno	Novigrad (Istria), Croatia	3-phases, Alfa-laval, 1500 kg/hour capacity	not filtered
IP_35	Croatia	Istarska bjelica (100%)	Novigrad (Istria), Croatia	3-phases, Alfa-laval, 1500 kg/hour capacity	not filtered
IP_36	Croatia	Rosinjola 100%	Novigrad (Istria), Croatia	3-phases, Alfa-laval, 1500 kg/hour capacity	not filtered
IP_37	Croatia	blend of Leccino (85%) and Pendolino (15%)	Novigrad (Istria), Croatia	3-phases, Alfa-laval, 1500 kg/hour capacity	not filtered
IP_38	Croatia	blend of Leccino (90%) and Pendolino (10%)	Novigrad (Istria), Croatia	3-phases, Alfa-laval, 1500 kg/hour capacity	not filtered, naturally sedimented and decanted
IP_39	Croatia	blend of Picholene (55%) and Lecio del Corno (45%)	Novigrad (Istria), Croatia	3-phases, Alfa-laval, 1500 kg/hour capacity	not filtered, naturally sedimented and decanted
IP_40	Croatia	Istarska bjelica	Novigrad (Istria), Croatia	3-phases, Alfa-laval, 1500 kg/hour capacity	not filtered, naturally sedimented and decanted
IP_41	Croatia	Oblica	Postira, island Brač, Croatia	2-phases, 1200 kg/hour	not filtered
IP_42	Croatia	Istarska bjelica	Vodnjan (Istria), Croatia	2-phases	filtered
IP_43	Croatia	Oblica	Primošten, Croatia	2-phases,2000 kg/hour capacity	filtered
IP_44	Croatia	Oblica	Primošten, Croatia	2-phases,2000 kg/hour capacity	not filtered
IP_45	Croatia	Buža puntoža	Umag (Istria), Croatia	2-phases, 500 kg/hour capacity	not filtered, naturally sedimented and decanted
IP_46	Croatia	Oblica	Postira, island Brač, Croatia	2-phases, 1200 kg/hour	not filtered
IP_47	Croatia	Oblica	Supetar, Island Brač, Croatia	3-phases	not filtered
IP_48	Croatia	Leccino	Island Pašman, Croatia	3-phases, 1000 kg/hour, first production process in the harvest year in olive mill	not filtered

IP_49	EU	Unknown	Unknown	Unknown	filtered
IP_50	Croatia	Leccino	Poreč (Istria), Croatia	2-phases, 2000 kg/hour capacity	filtered
IP_51	Croatia	Leccino	Poreč (Istria), Croatia	2-phases, 2000 kg/hour capacity	not filtered, oil left on the sediment
IP_52	Croatia	blend of Leccino (60%) and Istarska bjelica (40%)	Novigrad (Istria), Croatia	3-phases, Alfa-laval, 1500 kg/hour capacity	not filtered; naturally sedimented and decanted
IP_53	Croatia	Oblica	Postira, island Brač, Croatia	Hydraulic press, 300 kg capacity	not filtered
IP_54	Montenegro	Žutica	Bar, Montenegro	Stone mill and hydraulic press	not filtered
IP_55	Croatia	Oblica	Sutivan, island Brač, Croatia	Hydraulic press	not filtered
IP_56	Croatia	Oblica	Bobovišća, island Brač, Croatia	Hydraulic press	not filtered
IP_57	Greece	Koroneiki	Gargalianoi, Greece	2 phase, 6 tons per hour	not filtered
IT_15	Morocco	Unknown	Unknown	Unknown	Unknown
IT_16	Morocco	Unknown	Unknown	Unknown	Unknown
IT_17	Spain	P.D.O. Estepa - Hojiblanca	Spain	2-phases	filtered
IT_18	Spain	Picual, Picudo,Hojiblanca, Arbequina	Spain	2-phases	filtered
IT_19	Greece + Spain	Koroneiki, Arbequina, Picual, Cornicabra	Gargaliana, Pylos, Ilia, Ciudad Real, Cordoba, Badajoz	2-phases	filtered
IT_20	Spain	Unknown	Andalusia	2-phases	filtered
IT_21	Greece	Unknown	Greece	2-phases/3-phases	not filtered
IT_22	Greece	Unknown	Greece	2-phases/3-phases	not filtered
IT_23	Spain	Arbequina	Andalusia	2-phases	filtered
IT_24	Spain	Hojiblanca	Cordoba	2-phases	not filtered
IT_25	Spain	Picual	Jaen	2-phases	not filtered
IT_26	Tunisia	Chetoui, Chemlali	Tunisia	2-phases	not filtered

IT_27	Spain	Unknown	Catalonia	2-phases	not filtered
IT_28	Spain	Unknown	Andalusia	2-phases	not filtered
IT_29	Spain	Unknown	Andalusia	2-phases	not filtered
IT_30	Spain	Unknown	Cordoba & Jaen	2-phases	not filtered
IT_31	Greece	Unknown	Greece	2-phases	not filtered
IT_32	Spain	Hojiblanca	Cordoba	2-phases	not filtered
IT_33	Spain	Unknown	Sevilla, Murcia, Badajoz	2-phases	not filtered
IT_34	Spain	Unknown	Sevilla, Cadix, cordoba, Badajoz	2-phases	not filtered
IT_35	Spain	Unknown	Andalucia, Murcia, Bajadoz	2-phases	not filtered
IT_36	Tunisia	Unknown	Tunisia	2-phases	not filtered
IT_37	Spain	Arbequina	Extremadura	2-phases	not filtered
IT_38	Spain	Arbequina	Extremadura	2-phases	not filtered
IT_39	Spain	Arbequina	Extremadura, Cordoba, Sevilla, Murcia	2-phases	not filtered
IT_40	Unknown	Unknown	Unknown	Unknown	Unknown
IT_41	Unknown	Unknown	Unknown	Unknown	Unknown
IT_42	Unknown	Unknown	Unknown	Unknown	Unknown
UN_42	Italy	Main cv.: Leccino; Other: Frantoio, Moraiolo	Tuscany	3 phases; 2000 Kg/h	not filtered
UN_43	Greece	Unknown	Unknown	Unknown	Unknown
UN_44	Greece	Koroneiki	Malta, West Mani, Messinia	Hydraulic press. 1 ton/h	not filtered
UN_45	Greece	Manaki	Corinzia, Spathovouni	3 phases	not filtered
UN_46	Greece	Koroneiki	Stavropigio, West Mani, Messinia, Greece	2 phases: 4 tons/h	not filtered

UN_47	Italy	Nostrana di Brisighella P.D.O. Brisighella	Brisighella, Emilia-Romagna	3 phases	not filtered
UN_48	Greece	Koroneiki	Unknown	Unknown	filtered
UN_49	Unknown	Unknown	Unknown	Unknown	filtered
UN_50	Spain	Arbequina	Unknown	Unknown	filtered
UN_51	Greece	Unknown	Unknown	Unknown	filtered
UN_52	Italy	Unknown	Unknown	Unknown	filtered
UN_53	Tunisia	Sahli	Kairouan + Sousse + Nadhour + Mahdia	55% 3 phases; 45% 2 phases	not filtered
UN_54	Greece	Koroneiki	Peloponneso: Achladochori, Katsarou, Aalipsi Messini, Kalamata	83% 2 phases; 17% 3 phases	Filtered (01/02/2018 with fossil and cellulosic diatoms)
UN_55	Greece	Koroneiki	Malta, West Mani, Messinia	Hydraulic press. 1 ton/h	not filtered
UN_56	Spain	Hojiblanca	Sevilla	2 fasi, 200 T/24h	not filtered
UN_57	Spain	Picual	Montalban de Cordoba	2 phases	not filtered
UN_58	Greece	Koroneiki	Messimas	2 phases	not filtered
UN_59	Italy	Coratina, Ogliarola	Barletta Andria Trani, Apulia	Unknown	not filtered
UN_60	Italy	Coratina, Ogliarola	Barletta Andria Trani, Apulia	Unknown	not filtered
UN_61	Italy	Ogliarola / Coratina	Bari, Apulia	Prevaling 2 phases	not filtered
UN_62	Italy	Unknown	Lecce, Apulia	Prevaling 2 phases	not filtered
UN_63	Italy	Ogliarola / Coratina	Bari, Apulia	Prevaling 2 phases	not filtered
UN_64	Italy	Sinopolese	Reggio Calabria	3 phases	not filtered
UN_65	Italy	Coratina	Perugia, Umbria	2 phases	not filtered
UN_66	Greece	Main cv: Koroneki	Unknown	Prevaling 2 phases	not filtered

UN_67	Tunisia	Main cv: Chemlali	Unknown	Prevaling 3 phases	not filtered
UN_68	Italy	Moraiolo	Foligno, Umbria	3 phases	filtered
UN_69	Greece	Main cv: Koroneki	Unknown	Prevaling 2 phases	not filtered
UZ_28	Turkey	Memecik	Söke-Aydın-South Aegean	3-phases	unfiltered
UZ_29	Turkey	Memecik	Söke-Aydın-South Aegean	3-phases	unfiltered
UZ_30	Turkey	Memecik	Germencik-Aydın-South Aegean	3-phases	unfiltered
UZ_31	Turkey	Memecik	Kirazlı-Kuşadası-Aydın	2 -phases	unfiltered
UZ_32	Turkey	Gemlik-Memecik blend	Karacasu-Nazilli-South Aegean	3-phases	unfiltered
UZ_33	Turkey	Gemlik	Karacasu-Nazilli-South Aegean	3-phases	unfiltered
UZ_34	Turkey	Memecik	Germencik-Aydın-South Aegean	3-phases	unfiltered
UZ_35	Turkey	Memecik	Germencik-Aydın-South Aegean	3-phases	unfiltered
UZ_36	Turkey	Gemlik	Karacasu-Aydın	3-phases	unfiltered
UZ_37	Turkey	Memecik	Çine-Kızılgüney	3-phases	unfiltered
UZ_38	Turkey	Memecik	Kuşadası-Aydın	2 -phases	unfiltered
UZ_39	Turkey	Memecik	Torbali-Izmir	2 -phases	unfiltered
UZ_40	Turkey	Memecik	Torbali-Izmir	2 -phases	unfiltered
UZ_41	Turkey	Memecik	Torbali-Izmir	2 -phases	unfiltered
UZ_42	Turkey	Memecik	Kemalpasa-Izmir	2 -phases	unfiltered
UZ_43	Turkey	Saurani-Halhalı-Karamani blend	Antakya/Altınözü-South Anatolia	2 -phases	unfiltered
UZ_44	Turkey	Saurani-Gemlik Blend	Altınözü-Antakya	2 -phases	unfiltered
UZ_45	Turkey	Saurani-Gemlik Blend	Altınözü-Antakya	2 -phases	unfiltered

UZ_46	Turkey	Ayvalık-Edremit	Edremit-Balıkesir	2 -phases	unfiltered
UZ_47	Turkey	Ayvalık-Edremit	Edremit-Balıkesir	2 -phases	unfiltered
UZ_48	Turkey	Edremit-Domat-Gemlik blend	Akhisar-Manisa	2 -phases	unfiltered
UZ_49	Turkey	Memecik	Kemalpasa-Izmir	2 -phases	unfiltered
UZ_50	Turkey	Gemlik	Kemalpasa-Izmir	2 -phases	unfiltered
UZ_51	Turkey	Ayvalık-Edremit	Edremit-Balıkesir	2 -phases	unfiltered
UZ_52	Turkey	Unknown	Unknown	Unknown	unfiltered
ZRS_1	Slovenia	Istrska belica	Slovenia	2-phases	unfiltered
ZRS_2	Croatia	Istrska belica; Leccino, Buza	Slovenia	2-phases	filtered
ZRS_3	Slovenia	Blend of Istrska belica, Leccino and other varieties	Slovenia	2-phases	unfiltered
ZRS_4	Spain	Unknown	Unknown	Unknown	unfiltered
ZRS_5	Slovenia	Istrska belica	Slovenia	2-phases	filtered
ZRS_6	Slovenia	Blend of Istrska belica, Leccino and other varieties	Slovenia	2-phases	unfiltered
ZRS_7	Italy	Unknown	Unknown	Unknown	filtered
ZRS_8	Greece	Unknown	Unknown	Unknown	filtered
ZRS_9	EU	Unknown	Unknown	Unknown	filtered
ZRS_10	Mediterranean	Unknown	Unknown	Unknown	filtered
ZRS_11	Mediterranean	Unknown	Unknown	Unknown	filtered
ZRS_12	Mediterranean	Unknown	Unknown	Unknown	filtered
ZRS_13	Slovenia	Blend of Istrska belica, Leccino and other varieties	Slovenia	2-phases	unfiltered
ZRS_14	Slovenia	Blend of Istrska belica, Leccino and other varieties	Slovenia	2-phases	unfiltered

ZRS_15	Slovenia	Blend of Istrska belica, Leccino and other varieties	Slovenia	2-phases	unfiltered
ZRS_16	Slovenia	Blend of Istrska belica, Leccino and other varieties	Slovenia	2-phases	unfiltered
ZRS_17	Greece	Unknown	Unknown	Unknown	filtered
ZRS_18	Slovenia	PDO blend of Istrska belica, Leccino and other varieties	Slovenia	2-phases	filtered
ZRS_19	Mediterranean	Unknown	Unknown	Unknown	filtered
ZRS_20	Slovenia	Blend of Istrska belica and Leccino	Slovenia	2-phases	unfiltered
ZRS_21	Slovenia	Blend of Istrska belica and Leccino	Slovenia	2-phases	unfiltered
ZRS_22	Slovenia	Blend of Istrska belica, Leccino and other varieties	Slovenia	2-phases	filtered
ZRS_23	Croatia	Istrska belica, Leccino, Buža from Croatia	Slovenia	2-phases	filtered
ZRS_24	Italy	Peranzana	Slovenia	2-phases	filtered
ZRS_25	Croatia	Istrska belica, Leccino, Buža from Croatia	Slovenia	2-phases	filtered

Figure S1. Overlapping of the GC traces of extravirgin (EVOO) and lampante (LOO) samples and enlargement of some zones of chromatograms that seem more important for discriminating these two categories of samples.

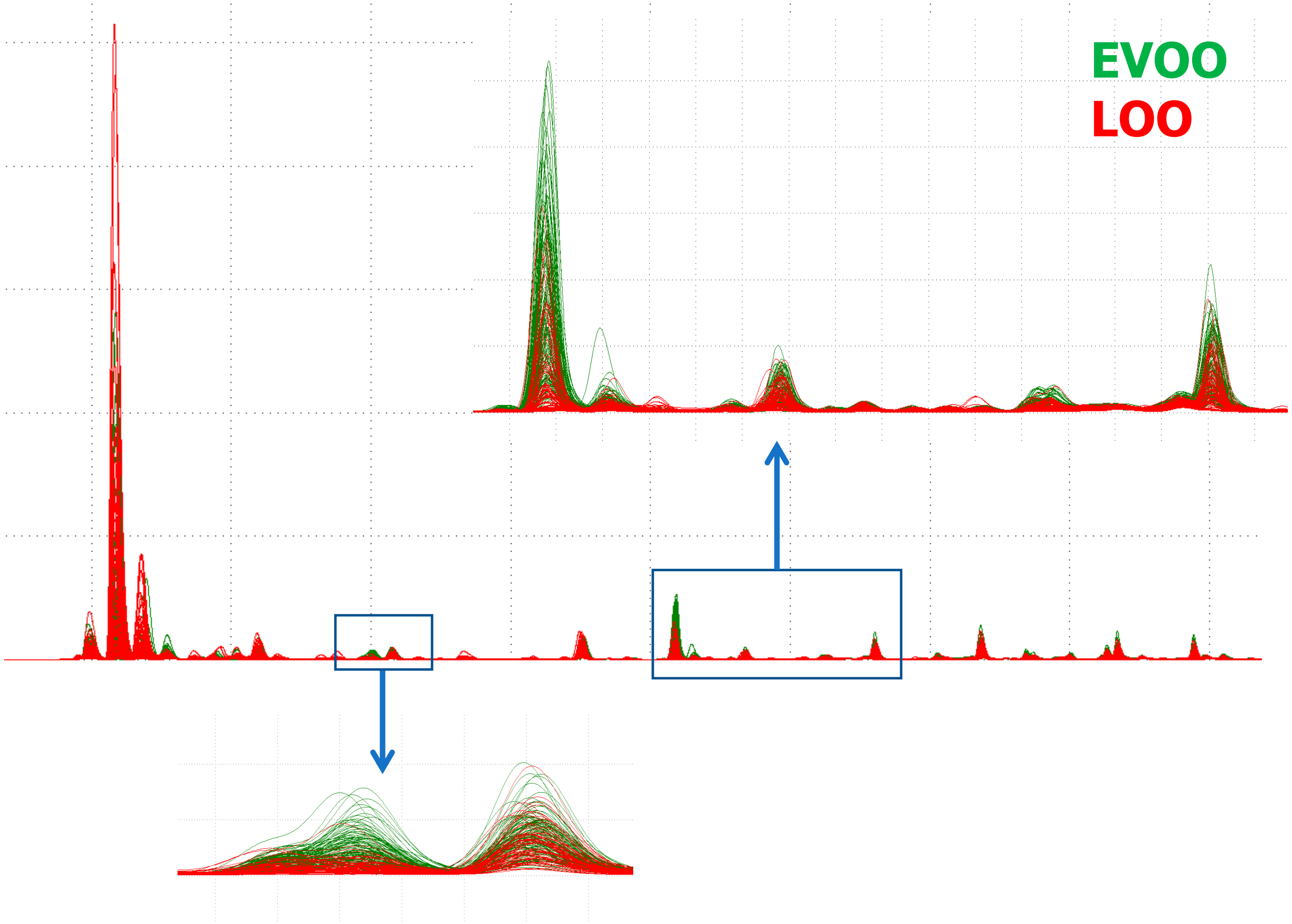

Supplement: Supplementary file 1 [file foods-09-00862-s001.pdf]
